# Cooperative learning–based family community education as a restorative justice strategy in juvenile diversion in Indonesia

**DOI:** 10.3389/fsoc.2026.1772801

**Published:** 2026-02-04

**Authors:** Adhani Wardianti, Uyu Wahyudin, Jajat Sudrajat Ardiwinata, Viena Rusmiati Hasanah, Hari Harjanto Setiawan

**Affiliations:** 1 Faculty of Education, Universitas Pendidikan Indonesia, Bandung, Indonesia; 2 National Research and Innovation Agency (BRIN), Jakarta, Indonesia

**Keywords:** children in conflict with the law, cooperative learning, family and community education, juvenile diversion, restorative justice

## 1 Introduction

Children in conflict with the law (CICL) constitute one of the most vulnerable groups within criminal justice systems worldwide. Beyond their legal status, these children frequently experience social stigma, psychological distress, disrupted education, and weakened family relationships—factors that significantly undermine their developmental trajectories and increase the risk of recidivism (Cavanagh, 2022). In response to these challenges, many jurisdictions have shifted away from punitive approaches toward diversion and restorative justice models that emphasize rehabilitation, accountability, and social reintegration rather than punishment.

Globally, restorative justice studies increasingly emphasize the central role of families as co-responsible and reintegrative actors, particularly in juvenile justice systems (Llewellyn, 2013; Kimbrell et al., 2023). However, across jurisdictions, a recurring challenge lies not in legal design, but in the limited relational readiness of families to engage meaningfully in restorative processes. Indonesia reflects this broader pattern, positioning family engagement as a normative ideal without consistently providing structured mechanisms for fostering it.

While adult education models such as andragogy and transformative learning offer valuable insights, cooperative learning is particularly well-suited to diversion contexts due to its relational and collective orientation. Unlike models that emphasize individual self-direction or critical awareness, cooperative learning prioritizes shared responsibility, peer interaction, and mutual support—core principles that closely align with restorative justice values. This makes cooperative learning highly adaptable for families facing collective diversion under conditions of stress and stigma.

Indonesia formally adopted this paradigm through Law No. 11 of 2012 on the Juvenile Criminal Justice System, which mandates diversion as a priority mechanism in handling juvenile cases. The law reflects a progressive restorative vision, positioning families and communities as key actors in supporting children's rehabilitation. Diversion is intended not merely as an alternative legal procedure but as a relational process that repairs harm, strengthens social bonds, and promotes the child's best interests (Sliva and Plassmeyer, 2021).

However, more than a decade after its enactment, the implementation of diversion in Indonesia continues to face substantial challenges. While the legal framework is well established, diversion practices at the community level often remain procedural and compliance-oriented. One of the most persistent gaps lies in the limited preparedness and engagement of families. Although families are legally recognized as central to diversion, many enter the process with minimal understanding of its principles, limited confidence in interacting with justice institutions, and inadequate psychosocial support (Joudrey et al., 2021). As a result, family participation is frequently passive, which weakens the restorative potential of diversion.

This article advances the position that diversion cannot succeed through legal reform alone. Restorative justice requires relational readiness—particularly within families—so that accountability, empathy, and behavioral change can be sustained beyond formal legal processes. Drawing on field-based experience and reflective analysis, we argue that cooperative learning–based family education offers a promising and underutilized strategy to address this gap.

Positioning family education as a restorative intervention rather than a supplementary activity reframes diversion as a lived social process. In doing so, cooperative learning aligns the normative goals of juvenile justice reform with the everyday realities of families navigating the diversion system. This Opinion article explores why such an approach is critical for strengthening diversion outcomes in Indonesia and offers broader reflections relevant to restorative juvenile justice efforts in comparable contexts.

This article does not propose family education as a formal legal instrument under the authority of the Ministry of Law and Human Rights. Instead, cooperative learning-based family education is positioned as a community-oriented pedagogical intervention that operates alongside formal diversion mechanisms. Its function is not to replace legal procedures, but to strengthen the relational and psychosocial capacities of families so that diversion can function as restorative justice in practice.

## 2 Diversion and the family engagement gap

Diversion is widely recognized as a cornerstone of restorative juvenile justice, intended to prevent the harmful consequences of formal criminal processing and to promote accountability in ways that are developmentally appropriate. At its core, diversion seeks to resolve cases through dialogue, responsibility-taking, and social reintegration, rather than punishment and exclusion (Wong et al., 2016). In the Indonesian context, this vision is explicitly articulated in the Juvenile Criminal Justice System Law, which places families at the center of the diversion process alongside community actors and justice professionals.

Despite this normative commitment, a persistent gap remains between policy intent and everyday practice. Diversion is often implemented as a procedural requirement rather than as a relational and rehabilitative process. Meetings may occur, agreements may be signed, and cases may be formally diverted, yet the deeper restorative goals healing, learning, and reintegration are not always realized. One of the key reasons for this shortfall is the limited engagement and preparedness of families, who are expected to play a central role without being adequately supported to do so.

Many families encounter the diversion process under conditions of stress, fear, and uncertainty. The involvement of law enforcement and legal institutions often evokes anxiety, particularly among families with limited legal literacy or prior negative experiences with state authorities. Social stigma surrounding juvenile offending further compounds this vulnerability, leading some families to withdraw from community life or to adopt a passive, compliance-oriented stance during diversion proceedings. In such contexts, families may attend meetings and follow instructions without fully understanding the purpose of diversion or their own agency within it.

This passive participation undermines the restorative logic of diversion. Restorative justice depends on meaningful participation, dialogue, and shared responsibility (Llewellyn, 2013). When families are unable or unwilling to engage actively—due to fear, confusion, or lack of confidence—diversion risks reproducing hierarchical and punitive dynamics under a restorative label. Importantly, this engagement gap is not a reflection of family disinterest or neglect, but rather a structural absence of family-centered preparation within diversion systems.

Recognizing this gap shifts the focus from individual family shortcomings to systemic design. If families are to function as restorative partners, diversion systems must invest in building their capacity, confidence, and understanding. This requires approaches that go beyond information delivery toward participatory and relational learning. It is within this context that cooperative learning–based family education emerges as a critical strategy for strengthening diversion as a genuinely restorative practice.

Evidence from diversion programs across jurisdictions suggests that limited family engagement is rarely due to individual unwillingness. Qualitative evaluations of diversion initiatives indicate that families often receive minimal preparatory support prior to engagement, resulting in formally compliant but substantively disengaged participation (Joudrey et al., 2021; Sliva et al., 2020). In Indonesia, diversion sessions are often held after procedural thresholds have been met, leaving little institutional space for relational preparation. This suggests that engagement gaps reflect systemic design limitations rather than a lack of family motivation.

## 3 Cooperative learning as a restorative family strategy

**Figure 1:**
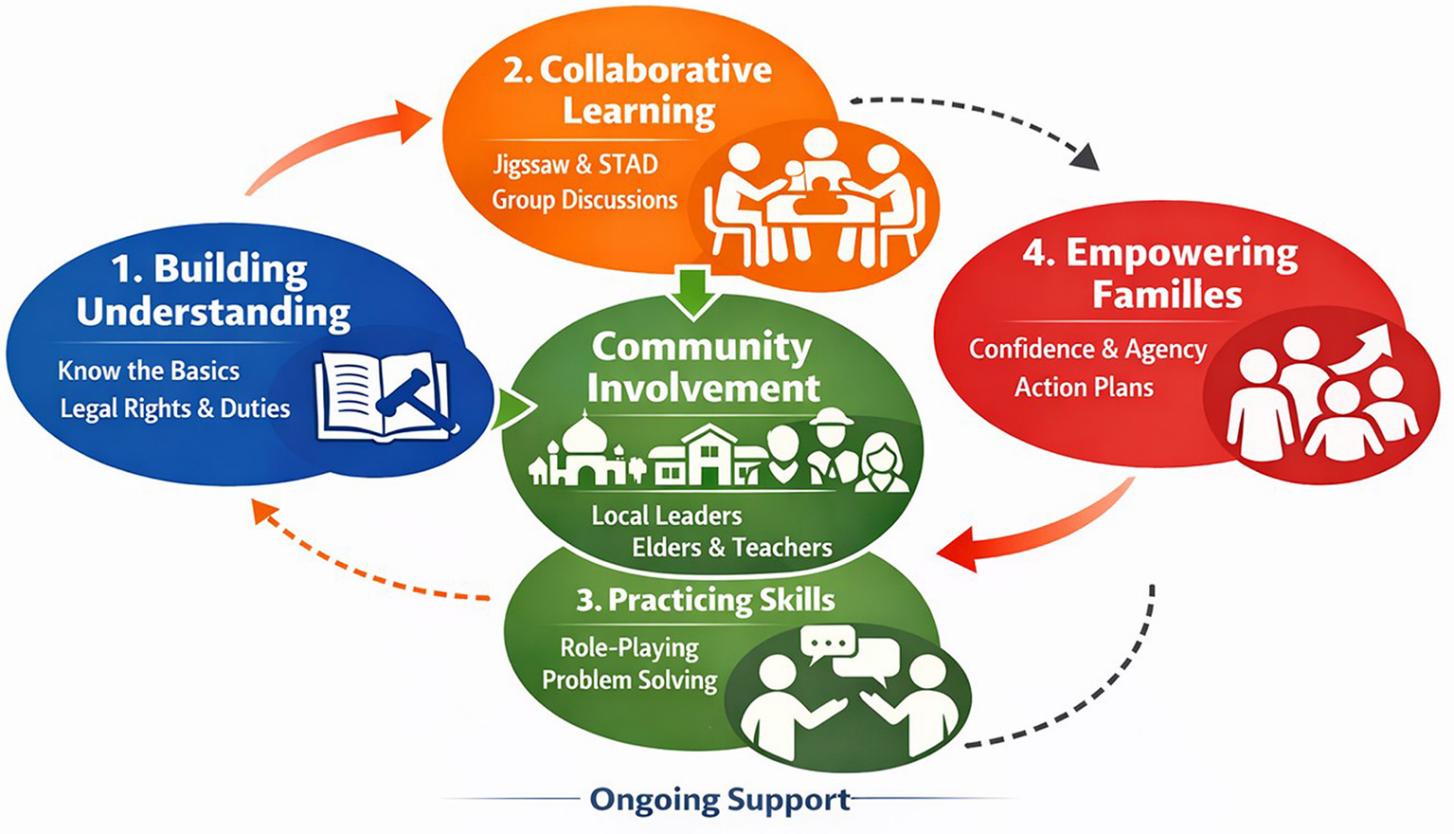
Cooperative learning process (family education steps in diverting child cases).

Restorative justice is fundamentally relational. It relies on dialogue, shared understanding, accountability, and collective responsibility rather than coercion or authority. For these principles to function in juvenile diversion, families must be equipped not only with procedural knowledge but also with the relational capacity to engage constructively with their children, victims, and justice system actors. Cooperative learning provides a pedagogical approach that directly supports these restorative requirements (Figure 1).

Cooperative learning emphasizes interaction, mutual dependence, and shared problem-solving among participants. Unlike traditional instructional models that position learners as passive recipients of expert knowledge, cooperative learning treats participants as co-constructors of meaning (Andersson et al., 2016). This orientation is particularly relevant in diversion contexts, where families bring lived experiences, emotional burdens, and contextual knowledge that cannot be addressed through one-way information transfer alone. By creating spaces for dialogue and peer exchange, cooperative learning allows families to collectively interpret diversion principles in ways that resonate with their social and cultural realities, thereby promoting successful reintegration of juvenile offenders (Thorpe et al., 2024).

As a restorative family strategy, cooperative learning offers several critical advantages. First, it normalizes participation by reducing isolation and stigma. Second, it fosters relational accountability by reframing family roles as shared responsibilities grounded in care and empathy. Third, it enhances practical competence through experiential activities such as role-playing interactions with justice officials or discussing real-life dilemmas. These processes build communication skills and confidence that are directly transferable to diversion contexts (Erickson, 2023).

Crucially, cooperative learning aligns with the restorative principle of empowerment. Families are not instructed on what to do; they are supported in discovering how they can act meaningfully within the diversion process. This shift from compliance to agency marks a significant transformation in how diversion is experienced. When families feel capable and respected, they are more likely to sustain engagement beyond formal diversion meetings and to reinforce rehabilitative values in everyday family life.

In the context of juvenile justice, cooperative learning thus functions as more than an educational technique. It operates as restorative infrastructure that strengthens the relational environment surrounding the child. By embedding cooperative learning within family education, diversion systems can move closer to their restorative ideals ensuring that accountability is accompanied by understanding, and that rehabilitation is supported by stable, informed, and engaged family networks.

In practice, cooperative learning in family education for case diversion can be operationalized through structured models such as Jigsaw and Student Teams Achievement Division (STAD). In the Jigsaw format, family members engage in small groups where each participant explores a specific aspect of case diversion—such as legal rights, communication strategies, or emotion regulation—and then shares insights with the group. This structure strengthens interdependence and shared understanding.

STAD-based activities can be used to foster collective problem-solving, for example, by discussing hypothetical case diversion scenarios and collaboratively identifying restorative responses. These models are intentionally simple, require minimal resources, and can be facilitated by probation officers, social workers, or trained community mediators, making them readily accessible to legal practitioners in the field.

Families involved in diversion often begin the process with confusion and stress, so the first step is Building Understanding: they are given the space to understand the basics of diversion, including their legal rights and obligations, so they no longer just “go along” without understanding. The process continues with Collaborative Learning, where families learn together through group discussions using models like Jigsaw and STAD; they share experiences, build collective understanding, and see that they are not alone in facing this situation. Understanding is then translated into action through Practicing Skills, which involves communication and resolution exercises through role-playing conflicts and problem-solving, so that families are ready to speak with authorities, mediate emotions, and support real changes in children's behavior. The final stage is Empowering Families, when families begin to gain confidence, are able to take an active role as restorative partners, and develop action plans for support at home and in the community. This entire series does not stop at one cycle, but is reinforced by ongoing support to ensure the changes are sustained and the child's reintegration is more stable.

In practice, cooperative learning models like Jigsaw and STAD can be expanded beyond configurations involving only families to include community participation. For example, community representatives can join study groups to discuss shared concerns about youth behavior, local norms, and collective responses to harm. This transforms diversion education into community-based restorative learning circles, rather than closed instructional sessions.

This configuration aligns with restorative justice practices that emphasize circles, shared storytelling, and multi-actor dialogue. While diversion processes in Indonesia may not always achieve the institutional complexity observed in formal reintegration or intake circles, even modest involvement of community actors can significantly shift relational dynamics from vertical to horizontal, strengthening the restorative nature of diversion.

## 4 Community as a restorative partner: moving beyond dyadic diversion

Restorative justice is fundamentally communal, not dyadic. While families play a crucial role in supporting children in conflict with the law, restorative processes risk reproducing hierarchical and therapeutic power relationships when engagement is limited to interactions between criminal justice authorities and the family itself. In such a dyadic configuration, state actors retain epistemic and moral authority, while families are positioned primarily as recipients of guidance or correction.

Community engagement serves as a necessary correction to this imbalance. In restorative justice theory, the community is not a passive backdrop but a moral stakeholder providing social norms, collective accountability, and reintegrative support (Llewellyn, 2013). The presence of community actors such as neighborhood leaders, religious leaders, educators, youth mentors, and local social organizations introduces horizontal relationships that reduce institutional dominance and transform deviance into a shared social process.

In cooperative learning-based family education, community actors can function as co-learners and facilitators, rather than as authority figures. Their participation in learning situations takes place within a social context shaped by class, ethnicity, and local power dynamics, which are particularly salient in juvenile justice encounters. Criminal justice interventions do not occur in socially neutral spaces; they are mediated by historical inequalities that shape trust, voice, and participation. Therefore, community inclusion enhances restorative legitimacy by grounding the diversion process in culturally resonant norms and shared responsibility.

When cooperative learning is embedded in a community setting such as a community center, religious institution, or neighborhood forum it enables families to shift from isolated compliance to collective engagement. Learning becomes relational not only within the family but also across social networks that ultimately support reintegration after formal diversion ends.

## 5 Reflections from field-based experience

Although this article does not present empirical findings in the form of a conventional research report, the arguments advanced here are grounded in sustained field-based experience with families involved in juvenile diversion processes. These reflections highlight recurring patterns that illustrate how cooperative learning–based family education can transform the lived practice of diversion.

The reflections presented here are drawn from the author's ongoing involvement in community-based diversion facilitation and family engagement activities in several urban and rural settings in Indonesia between 2019 and 2024. This experience included family assessments, participation in diversion meetings, family preparation sessions, and post-diversion follow-up conducted as a probation officer at the Bandung Correctional Center (Bapas Kelas I Bandung).

### 5.1 Reframing diversion from punishment to restoration

A consistent observation across diversion settings is that families initially approach the juvenile justice system with fear and defensive expectations. Legal processes are commonly perceived as punitive and adversarial, even when diversion is formally offered. This perception shapes family behavior, often resulting in minimal participation and a tendency to defer entirely to authority figures.

Within cooperative learning environments, families are gradually exposed to alternative interpretations of justice. Through dialogue, shared narratives, and guided reflection, diversion is reframed as a restorative process aimed at accountability, learning, and repair rather than punishment. When families begin to understand diversion as a pathway for growth, they are more willing to engage actively and to support rehabilitative goals at home.

For example, in one diversion case involving an 8 year old boy accused of injuring his friend's eye while playing, both families initially approached the process with fear and defensiveness. Although the incident was accidental, the seriousness of the injury and the involvement of the police created anxiety and social distance between the families. Initial mediation efforts failed because discussions focused solely on legal responsibility and compensation.

This dynamic changed only after community actors, specifically the neighborhood head (RT) and a local religious teacher, engaged in preparatory learning sessions with both families. Through facilitated, cooperative discussions in a familiar community setting, the families began to reinterpret the incident not simply as a violation of the law, but as a shared community concern involving child safety, supervision, and shared responsibility. This reframing reduced fear, softened conflicting positions, and opened space for restorative dialogue.

### 5.2 From passive caregivers to active restorative partners

Another recurring pattern involves changes in family roles. Prior to participatory education, caregivers frequently express uncertainty about how to communicate with police officers, counselors, or other justice professionals. This uncertainty often manifests as silence or compliance, which limits the family's contribution to diversion decision-making.

Cooperative learning activities—particularly peer discussions and role-playing help families develop confidence and practical communication skills. By practicing dialogue in a supportive environment, families learn how to express concerns, ask questions, and articulate their perspectives respectfully. Over time, this leads to a visible shift from passive attendance to active participation, with families positioning themselves as restorative partners in their children's rehabilitation.

During one diversion session, the perpetrator's family representative initially remained silent throughout the discussion with police officers, responding only when directly questioned. After participating in a cooperative learning session simulating a dialogue with justice actors, the family then initiated questions about the terms of the diversion agreement and rules. Such a shift illustrates how structured cooperative learning can translate into active restorative justice action.

In another case involving a 17 year old accused of distributing private digital content following a relationship conflict, the family initially remained silent during interactions with law enforcement, leaving the decision entirely to legal counsel and investigators. The parents expressed confusion and shame, particularly given the moral stigma associated with such offenses within their community.

After participating in cooperative learning sessions with community representatives, including local leaders and educators, the family developed greater confidence in articulating their perspectives. Role-playing exercises allowed them to practice restorative communication, not only with justice actors but also in anticipated dialogue with the victim's family and community members. This process transformed parents from passive observers into active restorative partners who could meaningfully support accountability and behavior change.

### 5.3 Community-embedded cooperative learning

Sustained rehabilitation depends not only on formal agreements but also on everyday relational practices within the family. A key reflection from field experience is that cooperative learning strengthens long-term family commitment to behavioral change. As families gain clarity about their roles and develop supportive networks, they are more likely to remain engaged beyond the conclusion of formal diversion meetings.

This commitment is reflected in increased emotional support, consistent supervision, and constructive communication within the household. Families begin to view rehabilitation as an ongoing relational process rather than a time-limited legal requirement. In this sense, cooperative learning bridges the gap between institutional diversion mechanisms and the lived realities of children's daily environments. Together, these reflections underscore a central insight: diversion becomes restorative only when families are prepared to engage relationally, emotionally, and practically. Cooperative learning–based family education offers a viable pathway for cultivating this readiness.

A further illustration can be seen in a violent incident involving a 17-year-old who acted impulsively in response to a perceived threat within his peer group. Although the offense resulted in serious harm and required formal legal intervention, the surrounding community neighborhood leaders, family members, and local institutions played a crucial role in preventing further escalation.

Cooperative learning activities were conducted not only with the family but also with selected community members who had social ties to both the perpetrator and the victim. These sessions emphasized shared norms, emotional regulation, and collective accountability. The inclusion of community voices helped position diversion as a communal response to adolescent conflict, rather than a purely institutional or therapeutic intervention.

## 6 Implications for policy and practice

The reflections presented in this article suggest that strengthening diversion outcomes requires a shift in how family engagement is conceptualized and operationalized within juvenile justice systems. If families are expected to function as restorative partners, policy and practice must move beyond symbolic inclusion toward meaningful capacity-building.

First, family education should be formally embedded within diversion procedures. Current diversion practices often assume that families will understand their roles once invited to participate, overlooking disparities in legal literacy, confidence, and psychosocial readiness.

Second, cooperative learning provides a scalable and culturally adaptable approach for community-based implementation. Because it relies on dialogue, peer support, and shared reflection rather than specialized infrastructure, cooperative learning can be integrated into existing community services, probation programs, or correctional centers.

Third, justice system actors require complementary training to support restorative family engagement. Police officers, probation counselors, social workers, and community leaders play a critical role in shaping families' experiences of diversion. Training in restorative communication and cooperative facilitation is essential to ensure coherence between family-centered interventions and institutional practices (Sliva et al., 2020).

Fourth, policy frameworks should recognize family engagement as an outcome in its own right. Diversion success is often measured narrowly through case resolution or recidivism rates, overlooking relational outcomes such as family confidence, communication quality, and sustained commitment to rehabilitation (Kimbrell et al., 2023).

Finally, future reforms should encourage cross-sector collaboration between justice institutions, educational providers, and community organizations. Cooperative learning thrives in environments where knowledge, experience, and responsibility are shared, enabling diversion systems to evolve into integrated restorative infrastructures.

Relational outcomes can be operationalized through qualitative and process-based indicators, such as documented family participation during diversion meetings, post-diversion follow-up interviews assessing communication quality, and facilitator assessments of family confidence and role clarity. These indicators need not replace recidivism metrics but can complement them, offering policymakers a more holistic understanding of diversion effectiveness. Integrating these indicators into probation reporting frameworks would be a significant step toward aligning policy evaluation with restorative justice principles.

## 7 Conclusion

This article has advanced the position that juvenile diversion cannot achieve its restorative objectives through legal frameworks alone. While diversion is formally embedded within Indonesia's juvenile justice system, its transformative potential depends fundamentally on the relational readiness of families to engage as active partners in rehabilitation. When families lack understanding, confidence, or psychosocial support, diversion risks being reduced to a procedural requirement rather than a meaningful restorative process.

Cooperative learning–based family education offers a practical and conceptually coherent response to this challenge. By emphasizing dialogue, shared learning, and collective responsibility, cooperative learning strengthens families' capacity to participate in diversion in ways that align with restorative justice principles. It supports a shift from passive compliance toward active engagement, enabling families to sustain rehabilitative values within everyday family life and community interactions.

However, cooperative learning reaches its full restorative potential only when it is embedded within community relationships. Without community participation, diversion can remain dyadic—structured primarily as an interaction between justice authorities and families—thereby risking hierarchical and therapeutic dynamics. When community actors such as neighborhood leaders, educators, faith-based figures, and local organizations are included as co-participants, diversion becomes a shared moral and social process that distributes responsibility, reduces stigma, and strengthens reintegration.

In conclusion, embedding cooperative learning–based family education within community-supported diversion practices can help transform diversion from a legal alternative into a relational system of care. Such an approach strengthens accountability, promotes healing, and enhances prospects for sustainable reintegration of children in conflict with the law in Indonesia and in other contexts where restorative ideals are challenged by limited family and community readiness.
